# Inferred network from prefrontal cortex activity of rats unveils cell assemblies

**DOI:** 10.1186/1471-2202-14-S1-O20

**Published:** 2013-07-08

**Authors:** Gaia Tavoni, Ulisse Ferrari, Francesco Paolo Battaglia, Simona Cocco, Rémi Monasson

**Affiliations:** 1 Laboratory of Statistical Physics, CNRS & École Normale Supérieure, Paris, France; 2 Laboratory of Theoretical Physics, CNRS & École Normale Supérieure, Paris, France; 3 Donders Centre for Neuroscience, Nijmegen, The Netherlands

## 

We analyzed recordings of prefrontal cortex activity of a rat in three different phases: while the animal faces a task in which a rule has to be learned and during the previous and subsequent sleep phases. We inferred an Ising model (characterized by binary variables and local fields and couplings as parameters) from the recorded spiking frequencies and pairwise correlations between neurons. We have shown how the inferred model can be used to deepen the analysis of the recordings, unveiling the presence of highly coordinated groups of neurons (cell assemblies), that is neurons that are activated together and synchronously inhibit the activity of other specific neurons.

**Figure 1:**
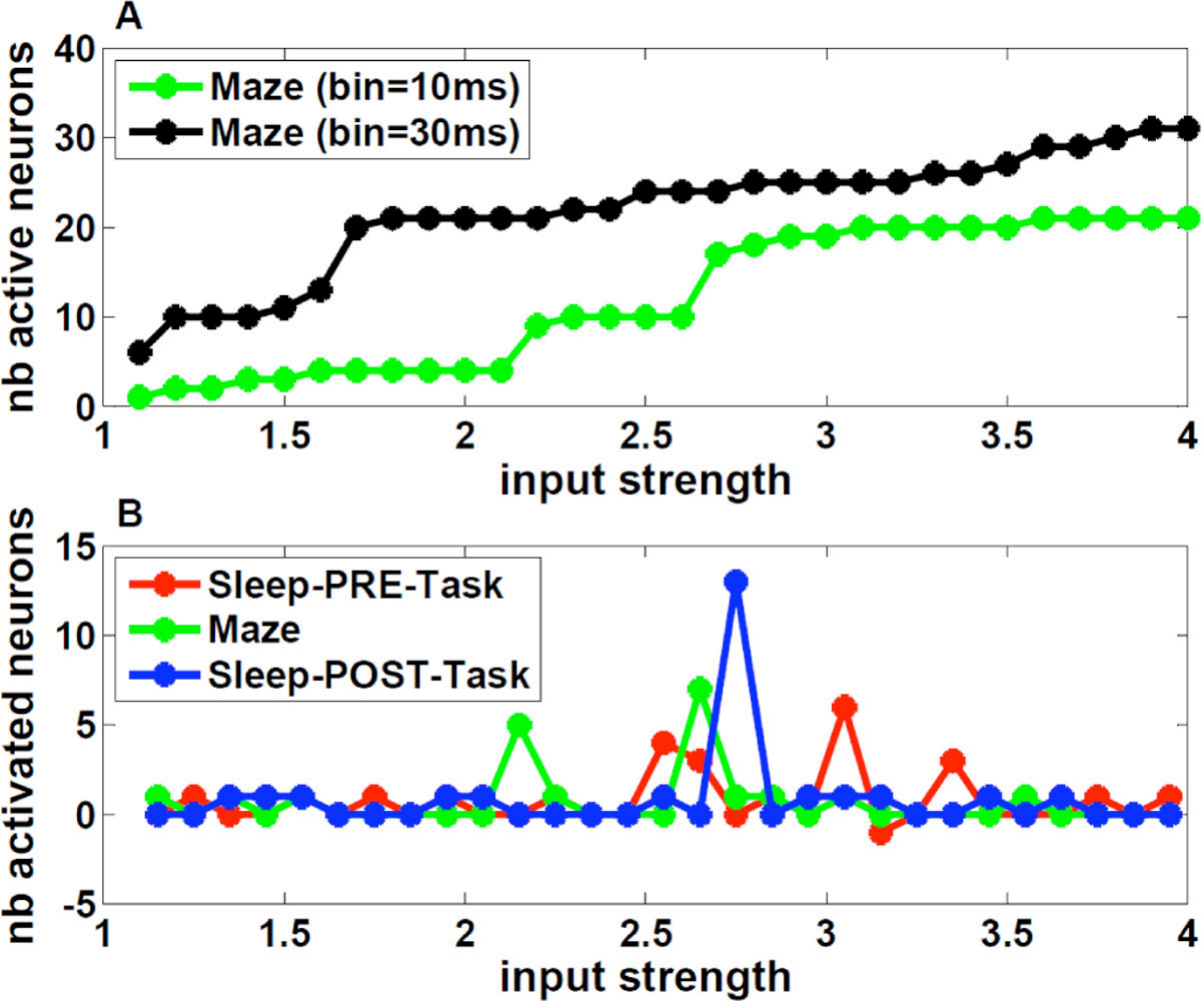
**Co-activation of neurons as a function of the input strength and of the time bin**. **A**: Number of active neurons A(H) in the stable states vs external input H. Parameters (fields and couplings) of the Ising model were inferred from the activity of the Maze epoch, binned into time windows of Δt1 = 10 ms (green) and Δt2 = 30 ms (black). The two curves can be superimposed upon translation of the input strength by log(Δt2/Δt1). **B**: Number of newly activated neurons, defined through A(H +0.1)-A(H), in the stable configurations for the three epochs vs. input strength H; the fields and couplings of the Ising model have been inferred for each epoch.

To identify the coactivated groups, we found the maxima of the log-likelihood of a configuration of neurons (stable states), defined as the sum of all the fields and couplings relative to the active neurons, performing an ascent dynamics on the energy landscape. When the model is inferred from the activity binned into 10 ms time bins, the only stable state is the one with all silent neurons. By adding an external input into the model and slowly increasing its value, stable states with more and more active neurons appear (see Figure 1A), starting from the neurons with higher spiking frequency. Remarkably, the curves in Figure 1A show large jumps at specific values of the input strength, corresponding to the co-activation of strongly interconnected neurons, which not necessarily have high average activity. These highly synchronized neurons have been found from the models of both the awake and sleep epochs (see Figure 1B), and are partially shared between different phases.

We investigated the meaning of the external input parameter, discovering that it carries information on the time scale at which we observe correlations between neurons, namely the time bin width. In fact, the two curves of Figure 1A, which refer to the model inferred from the neuronal activity binned into two different time bins (10 ms and 30 ms), overlap by applying a translation of log(30 ms/10 ms) in the input strength. The fact that at Δt = 30 ms the first co-activated group appears for a small input strength H~1 means that the group is likely to be co-activated in a 30 ms time scale.

Neurons found in activated and inhibited groups extracted from our model correspond to large entries in the two principal eigenvectors of the Pearson correlation matrix obtained from the recorded activity. In particular the 1st component has large and positive entries on both activated groups, and the 2nd component shows negative entries on the 1st group, and positive ones on the 2nd group which entails that the groups can activate together or not. Moreover the activation of a group causes the inhibition of another group, which has also large entries on the 1st and 2nd components but with opposite signs. The sign of the components therefore refects in an intricate manner the activation-inhibition relationships between different groups.

